# Surgical Treatment of a Medial Clavicle Fracture Nonunion with Medial Clavicle Resection and Stabilization to the Sternum with Palmaris Longus Graft

**DOI:** 10.1155/2019/7123790

**Published:** 2019-10-24

**Authors:** M. O. Dion, S. Martel, S. Pelet

**Affiliations:** ^1^Centre de Recherche FRQS du CHU de Québec—Hôpital Enfant-Jésus, 1401, 18ème Rue, Québec, Québec, Canada G1J 1Z4; ^2^Department of Orthopedic Surgery, CHU de Québec—Hôpital Enfant-Jésus, 1401, 18ème Rue, Québec, Québec, Canada G1J 1Z4

## Abstract

Medial end clavicular fractures are a rare occurrence. While most of these fractures can be appropriately managed with a nonoperative treatment, some cases of symptomatic nonunion might be surgically addressed to preserve sternoclavicular joint stability and ensure favorable outcomes. The open reduction and osteosynthesis procedure is a commonly performed procedure to treat clavicular fracture nonunion. However, few revision procedures have been described to address the occasional cases of hardware failure or recurrent nonunion of the medial end. In this report, the authors present a case of symptomatic nonunion of the medial clavicle initially treated with osteosynthesis. Implant failure with hardware migration was then treated by medial clavicle resection and stabilization to the sternum using a *palmaris longus* autograft and the figure-of-eight lacing technique. Excellent functional outcomes at three years of follow-up were obtained. To the authors' knowledge, this is the first case reporting on a sternoclavicular stabilization with a tendon autograft for such an important bone deficit.

## 1. Introduction

Medial end clavicle fractures are the least prevalent type of clavicular fractures. Accounting for 2 to 3% of all clavicular fractures, they can frequently be appropriately managed with a nonoperative treatment [1, 2]. However, symptomatic nonunion occurs in about 8% of medial clavicular fractures [2]. In those cases, surgical management becomes a valued approach to obtain clavicular consolidation, prevent lasting pain, and restore normal shoulder function in patients. Currently, there is no consensus on the best surgical approach to manage medial clavicular fracture nonunion. Open reduction and internal fixation (ORIF) with plates and screws has shown fair to good outcomes to treat medial clavicle factures and symptomatic nonunions [3, 4].

Multiple complications have been reported with medial clavicle ORIF, with one of the most disabling being metal migration [5]. Stiff reconstructions of the sternoclavicular (SC) joint create high bending forces dissipated through the construct during movement of the shoulder girdle and clavicle. This factor contributes to the high failure and metal migration rates of stiff constructs. In addition, the inherent difficulty to stabilize the small medial bone fragment of the clavicle makes it hard to obtain a proper fixation [3]. These two factors can contribute to plate loosening following ORIF procedures. Managing implant failure is challenging for surgeons, as few procedures are described for revision. Although initially proposed for the treatment of SC instability, medial partial claviculectomy is now being proposed to treat medial clavicular nonunion [6].

## 2. Case Report

**Figure 1:**
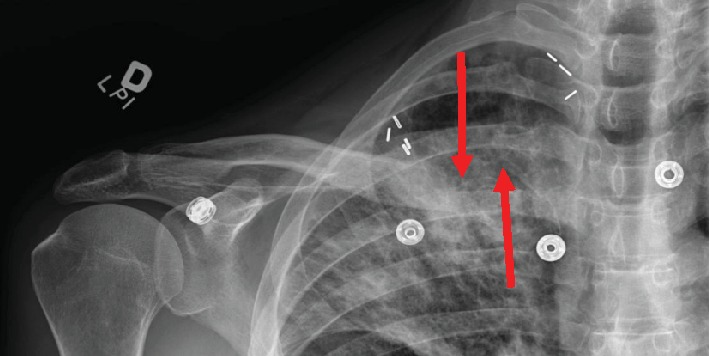
Clavicle radiograph five months after the initial injury showing a right medial clavicle fracture nonunion (red arrows). Vascular clips are from a rib resection at a young age.

**Figure 2:**
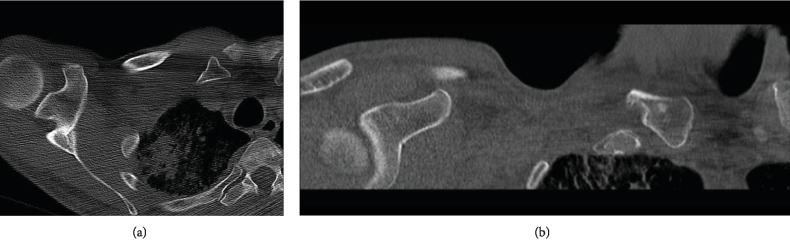
CT images five months after the initial injury showing a right medial clavicle fracture nonunion (a) with sclerotic changes of the medial clavicle fragment (b).

A 55-year-old man was referred from a regional hospital to a university hospital (level-one trauma center) for symptomatic nonunion of a right medial clavicle fracture that resulted from a fall injury. The patient was a smoker and was known for left reverse total shoulder arthroplasty, first right rib resection at a young age, bipolar disorder, and antisocial personality disorder. His baseline level of activity was low, as he was unemployed and did not practice a specific sport or physical activity. His range of motion of the left shoulder was limited due to his previous reverse total shoulder arthroplasty. When the patient was first evaluated at the outpatient clinic 5 months after the injury, the active range of motion of his right shoulder was reduced in all three planes to about 70 degrees in abduction, 90 degrees in flexion, and 30 degrees in external rotation due to pain and medial instability. The abduction strength was 4+ at the Jobe test. The primary complaint of the patient was not pain, but the sensation of instability at the fracture site during movement. Radiographic and CT imaging demonstrated a highly comminuted medial-end nonunion of the right clavicle (Figures 1 and 2).

**Figure 3:**
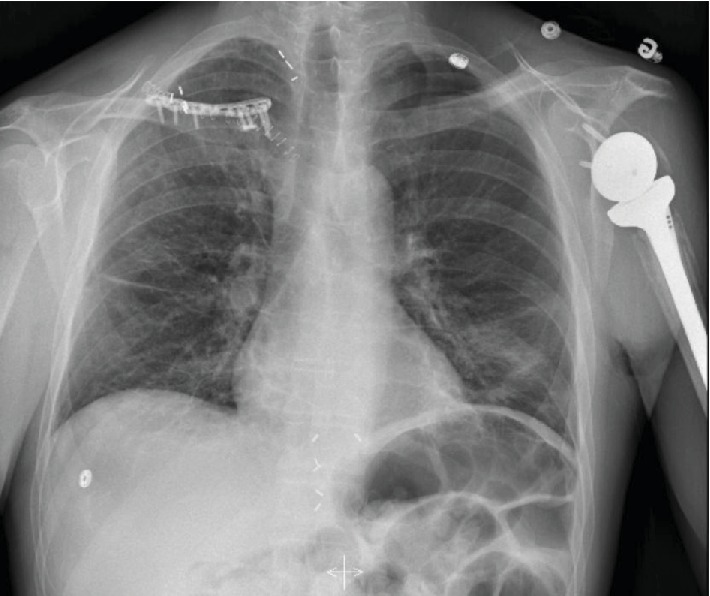
Postoperative chest radiograph following the open reduction and osteosynthesis procedure. The fracture was fixed using an inverted anatomic locking plate.

Surgical management of the nonunion with an open reduction and internal fixation was performed. In a semisitting position, the nonunion site was opened using a 10-centimeter longitudinal incision at the SC joint. ORIF was completed using a locking clavicle distal-end plate (Stryker, Mahwah, NJ) and a 3.5 mm lag screw. A left-side plate was positioned so that the larger portion (usually lateral portion) could fit on the clavicular medial end. One lag screw was used to maintain fracture reduction. Four locking screws were used on the medial side of the fracture, and three locking screws were used on the lateral side (Figure 3). The SC joint was not bridged by the plate, and a stable fixation was obtained. Two weeks of immobilization in a sling were recommended to the patient, but it was suspected that his compliance to the proposed treatment was inadequate.

**Figure 4:**
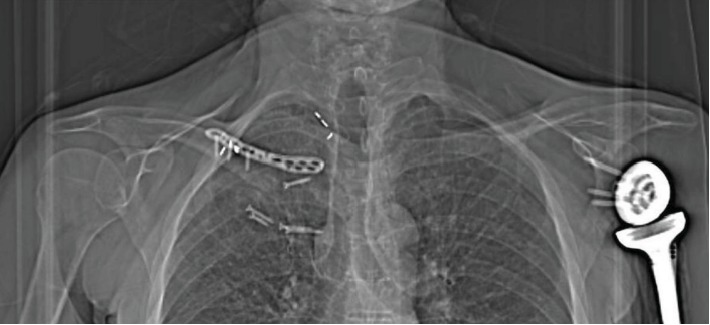
Five months post-ORIF. Scout view of a CT scan illustrating hardware migration and displaced comminuted bone fragments.

**Figure 5:**
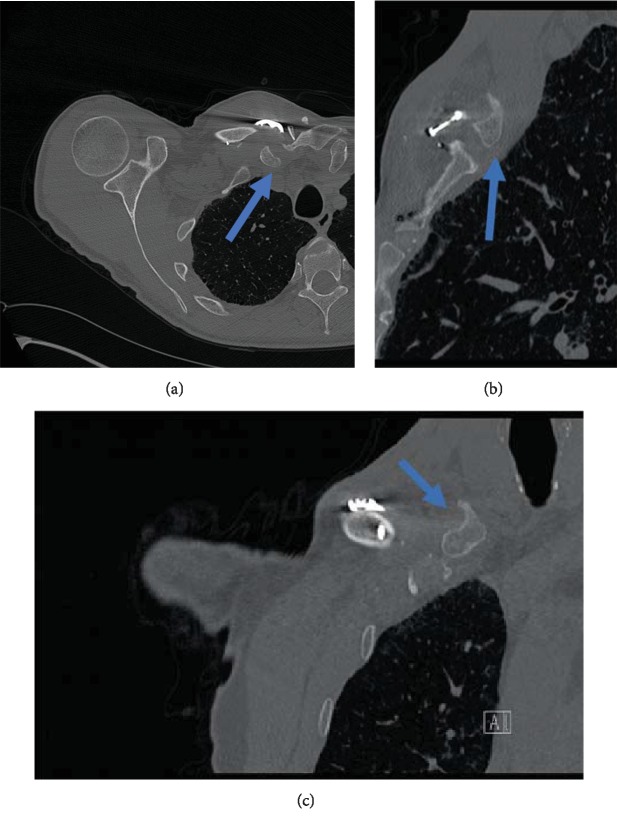
Five months post-ORIF. CT images showing hardware migration and posterior displacement of the remaining medial end of the right clavicle (blue arrow) (a–c).

During a follow-up appointment at his regional hospital five months postoperatively, the patient complained of increasing pain and instability at the SC joint. This injury limited the patient in some of his activities of daily living, such as dressing and personal hygiene. The wound healing seemed complete, and there was no sign of operative site infection. A new series of radiographs showed extensive plate loosening and migration of the five medial screws (4 locking+1 lag screw). Two medial locking screws were now over 3 centimeters away from the plate. The three distal screws remained properly positioned. The patient was referred to the university hospital for further evaluation. A CT scan of the shoulder and the affected SC joint confirmed hardware migration, displacement of the comminuted bone segments, and posterior displacement of the medial fragment of the clavicle with the SC joint still intact (Figures 4 and 5). A medial claviculectomy with stabilization of the remaining clavicle was proposed.

Under general anesthesia and in a semisitting position, the same longitudinal skin incision was opened to access the nonunion site. The plate and the three intact distal screws were removed. Three loose medial screws were removed, but two medial screws (Figure 4) remained unreachable. The comminuted bone fragments were removed, and the medial end of the right clavicle was resected for a total of 5 centimeters in length. Two 6-millimeter tunnels were drilled on both the sternum and medial clavicle in preparation for the figure-of-eight reconstruction technique. The tunnels were drilled directly through the medial end of the clavicle, while they were drilled with an oblique trajectory on the sternum from the anterior cortex towards its right articular surface. The patient's left *palmaris longus* tendon was harvested as the autograft of choice for this surgery, as the right *palmaris longus* was absent. A good quality, 15-centimeter-long *palmaris longus* tendon was obtained. The graft was passed through both tunnels in a figure-of-eight configuration and sutured near the SC joint (no pretightening). Both free ends were sutured to one another using Ethibond 1-0 (as described by Bae et al. [7]). Vicryl 0 sutures were used for subcutaneous closure, and staples were used for skin closure. Two weeks of immobilization were recommended to the patient with a shoulder sling.

**Figure 6:**
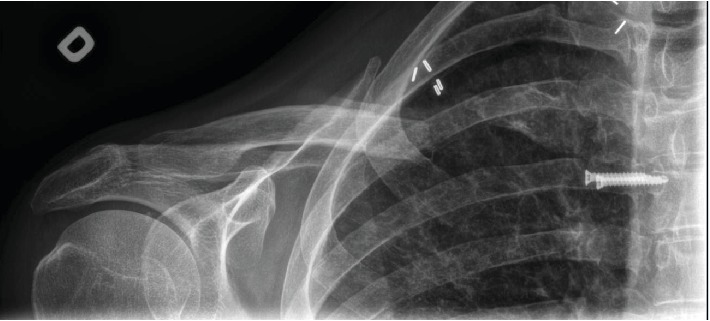
Clavicle radiograph three years postreconstruction surgery. Two remaining screws are superficial to the sternum.

Surgery was well tolerated by the patient. Cultures taken during revision surgery remained negative. Wound healing was uneventful. Two weeks after surgery, the patient presented to the clinic for evaluation. He reported minimal pain and had a range of motion of approximately 150° in flexion and abduction. Eight months postoperatively, the patient did not present any residual pain or limitation on range of motion. Outcomes at 3 years of follow-up were excellent, with no range of motion limitation, no pain, and no clinical instability. Control radiographs showed no sign of reconstruction failure (Figure 6). The patient was able to return to his previous level of activity and was able to perform his activities of daily living without any limitation.

## 3. Discussion

Although surgical management of symptomatic medial clavicle nonunion with an ORIF usually yields high consolidation rates and good functional outcomes [4], this technique resulted in hardware migration and nonunion in the reported case. Many factors may have contributed to the osteosynthesis failure, including the patient's poor compliance to the immobilization treatment, the lack of a costoclavicular ligament to stabilize the medial clavicle due to a past first right rib resection, and the fact that the patient was a smoker.

This original case report outlines the benefits of a less familiar technique to stabilize the SC joint when a large gap is created after a substantial medial clavicle resection. Since the options for revision surgery were limited by the highly comminuted nature of the fracture, we opted to excise the nonunion site of the medial clavicle, followed by the stabilization of the SC joint with a tendon autograft placed using the figure-of-eight technique. The rationale behind this procedure was to remove the nonunion site and to treat the remaining deficit as SC instability, with the figure-of-eight suture and fixation technique resulting in a more stable (mainly vertical) procedure than a single loop, and preventing early failure by reducing graft-to-bone friction. Since resection of the medial clavicle without reconstruction has been associated with poor functional outcomes in previous studies [8], stabilization of the clavicle to the sternum allowed for a better functional outcome. This type of stabilization reduces the instability of the residual medial clavicle and prevents axial displacement of the clavicle towards the shoulder. However, this type of reconstruction does not prevent axial displacement of the clavicle towards the sternum, thus allowing a certain degree of residual instability in comparison to the anatomic SC articulation. In the reported case, the patient did not complain of residual shoulder instability following the procedure. It is possible that the adaptation over the years to the lack of costoclavicular ligament stabilization allowed the patient to better tolerate the mild residual axial instability that would result from this type of procedure.

Symptomatic SC instability is well described in the literature. Several surgical procedures have been described to treat this condition [9–13]. Soft tissue reconstruction of the SC joint with or without a resection of the medial clavicle is a popular choice for the treatment of chronic SC instability. However, current tendon autograft reconstructions using the figure-of-eight technique are described with an intact or slightly shortened clavicular medial end. Studies suggest that the medial clavicular resection should not exceed 1.5 centimeters in order to preserve the function of the costoclavicular ligament and maintain SC joint stability [9]. In contrast, this report presents a case where stabilization of the SC joint with a bone deficit of 5 centimeters was achieved with favorable functional results despite the absence of a functional costoclavicular ligament. To our knowledge, this is the first case in the literature reporting SC stabilization with a tendon autograft for such a large bone deficit.

The figure-of-eight reconstruction was deemed the technique of choice for the reported procedure. This reconstructive method was described as the stiffest reconstruction surgery for SC instability repair in cadaveric studies with intact medial clavicles [14]. Its effectiveness in vivo was also validated through a series of recent studies and reports [15]. In addition, the senior author (SP) was familiar with the figure-of-eight reconstruction technique. The sternal tunnels were drilled in an oblique direction in order to limit retrosternal dissection and eliminate the need for anteroposterior drilling through the manubrium, consequently minimizing the risks of significant intraoperative complications [16]. The availability and length of the tendon graft required to bridge the remaining bone deficit is an important limiting factor for this type of reconstruction. The use of various tendon grafts has been reported for the reconstruction of the SC joint (*semi-tendinosus*, *gracilis*, *palmaris longus*, *sternocleidomastoid*, and *subclavius*). In this case, the *palmaris longus* was selected because its harvest is simple and presents low harvest site morbidity [17]. Even if the length and cross-sectional diameter of the *palmaris longus* can be near the inferior limit for this type of procedure [18], the 15-centimeter autograft obtained was long and bulky enough for the reconstruction.
